# Effects of Low-Volume Static Stretching and Full Range-of-Motion Resistance Training on Flexibility in Physically Active Young Adults: A Randomized Controlled Trial

**DOI:** 10.3390/jcm15155783

**Published:** 2026-07-23

**Authors:** Michał Kaczorowski, Karol Jaskulski, Kamil Góra, Sebastian Usiądek, Wiktoria Pytko, Ida Wiszomirska

**Affiliations:** 1 Faculty of Rehabilitation, Jozef Pilsudski University of Physical Education in Warsaw, Marymoncka 34, 00-968 Warsaw, Poland; karol.jaskulski@awf.edu.pl (K.J.); su78461@stud.awf.edu.pl (S.U.); wp79175@stud.awf.edu.pl (W.P.); ida.wiszomirska@awf.edu.pl (I.W.); 2 Independent Researcher, Motorowa 1, 04-035 Warsaw, Poland; fizjokultura@gmail.com

**Keywords:** mobility, time-efficient training, stretching exercise, strength training, minimal-dose

## Abstract

**Background:** The purpose of the present study was to compare the effects of a low-volume static stretching protocol and low-volume full range-of-motion resistance training on flexibility in physically active young adults. **Methods:** A total of 48 participants (18–25 years) were included in the final analyses. Participants were randomly allocated to a static stretching group (SS, *n* = 16), a resistance training group (RT, *n* = 16), or a control group (CG, *n* = 16). The intervention lasted six weeks and consisted of one supervised session per week. The SS group performed two 60 s sets of high-intensity static stretching, whereas the RT group completed full range-of-motion exercise with a total duration equivalent to that of the SS group. **Results:** Significant improvements in flexibility (pre-to-post change) were observed in both intervention groups compared with the control group. The SS group demonstrated a statistically significant increase in range of motion compared with the control group (3.93 ± 3.45 cm, 95% CI: 2.09–5.77 vs. −0.25 ± 0.93 cm, 95% CI: −0.75–0.25, *p* < 0.001; Cohen’s d = 1.65), while the RT group also improved significantly compared with the control group (1.81 ± 1.75 cm, 95% CI: 0.88–2.74 vs. −0.25 ± 0.93 cm, 95% CI: −0.75–0.25; *p* = 0.039; Cohen’s d = 1.47). **Conclusions:** Both low-volume static stretching and full range-of-motion resistance training performed once weekly for six weeks effectively improved posterior chain flexibility in physically active young adults.

## 1. Introduction

Increases in flexibility following acute static stretching (i.e., a single static stretching session) are commonly observed immediately after a single session of static stretching. However, these effects appear to be short-lived. A meta-analysis performed by Konrad et al. (2021) revealed that the increase in joint range of motion induced by one bout of static stretching is retained for approximately 20 min following the intervention [1]. Despite the consistently reported improvements in flexibility following both acute (i.e., a single stretching session) and chronic (i.e., repeated stretching sessions over time) static stretching, the exact physiological mechanisms underlying these adaptations remain unclear [2]. Current evidence suggests that enhanced flexibility may be related to mechanical and structural alterations within the muscle-tendon complex, in addition to increased stretch tolerance driven by neural adaptations [3,4,5,6,7]. In a systematic review, Ingram et al. [2] investigated the mechanisms responsible for range-of-motion improvements following acute and long-term static stretching interventions. Their findings indicated that chronic stretching decreases overall musculotendinous stiffness and increases stretch tolerance regardless of stretching intensity, intervention duration, sex, or initial flexibility status. By contrast, acute static stretching reduced stiffness only in individuals presenting normal flexibility levels, provided that stretching was performed at moderate or high intensity, while no concomitant increase in stretch tolerance was identified. Furthermore, no alterations in fascicle length were observed after either acute or chronic stretching interventions. Collectively, these findings suggest that long-term increases in range of motion are predominantly associated with reductions in stiffness and enhanced stretch tolerance.

The effectiveness of various exercise interventions, particularly different forms of stretching, for improving range of motion has been confirmed across numerous studies [8,9,10]. Additionally, a meta-analysis conducted by Thomas et al. (2018) demonstrated that static stretching elicits significantly greater improvements in flexibility compared with ballistic stretching and proprioceptive neuromuscular facilitation (PNF) [8]. Conversely, Medeiros et al. (2017) reported comparable effectiveness among static, ballistic, and PNF stretching techniques in improving ankle dorsiflexion range of motion [11]. Similarly, Konrad et al. (2023) showed that static stretching and PNF produce comparable increases in range of motion, with both methods demonstrating superior effectiveness relative to ballistic and dynamic stretching approaches [12]. Static stretching appears to be an optimal method for increasing range of motion, as alternative approaches have demonstrated comparable or less favorable outcomes. Research investigating the effects of static stretching on range of motion has primarily focused on identifying the optimal training variables, including type of stretching, duration, frequency, and intensity. A meta-analysis published in 2024, including 189 studies and a total of 6654 adults, demonstrated that the optimal dose of static stretching for long-term improvements in range of motion is approximately 10 min of static stretching per muscle group per week [13].

Previous research has also demonstrated that resistance training may increase range of motion with effectiveness comparable to that of static stretching. In their meta-analysis, Jootier et al. [14] analyzed 11 studies, eight of which reported similar improvements in flexibility in both the resistance training and static stretching groups. Importantly, weekly training volume, expressed as total training time per week in minutes, was not reported in the majority of the included studies. In a study by Rosenfeldt et al. [15], full range-of-motion resistance training was compared with static stretching. Both intervention groups completed supervised training sessions with matched planned active exercise duration, while each intervention was performed at a high intensity over an eight-week period. Training volume progressively increased throughout the intervention, from 6 min and 24 s per week during the first week to 12 min and 48 s per week during the final week. Both intervention groups demonstrated improvements in range of motion, with no significant between-group differences. It should be emphasized that during five of the eight intervention weeks, participants performed a weekly training volume considered optimal for increasing posterior chain flexibility.

The increasing number of studies investigating the optimization of training volume and other static stretching variables for enhancing range of motion is particularly important for individuals aiming to achieve the greatest possible improvements in flexibility within the shortest feasible timeframe. Nevertheless, it is important to consider that a substantial proportion of the population remains physically inactive. In Poland, it is estimated that as many as 43% of adults do not engage in any form of physical activity, compared with 54% in the USA [16,17]. Among this population, the most frequently cited barriers to initiating physical activity include lack of time, the perception of exercise as being too difficult, and insufficient access to equipment [18,19]. It is plausible that short, time-efficient, and effective exercise protocols could help increase physical activity participation among sedentary individuals while also providing an additional practical tool for improving flexibility. The concept of a minimal effective dose is well documented in the context of resistance and cardiovascular training [20,21,22,23]. In flexibility training, the concept of low-volume, time-efficient static stretching protocols is not yet well understood. To date, there appears to be a lack of research investigating the effectiveness of low-volume static stretching interventions and low-volume full range-of-motion resistance training interventions for improving range of motion.

Although insufficient time is frequently cited as a barrier to exercise participation in the general population, time-efficient exercise strategies are also highly relevant for physically active individuals. Athletes and recreationally active adults must integrate flexibility training with resistance training, sport-specific practice, conditioning, and recovery. Consequently, interventions that improve flexibility while requiring minimal additional training time are of considerable practical value. From this perspective, evaluating whether meaningful improvements in flexibility can be achieved with low-volume stretching protocols may help optimize training efficiency and facilitate the incorporation of flexibility training into already demanding training schedules.

We hypothesized that low-volume static stretching and low-volume full range-of-motion resistance training, performed once weekly for six weeks, would produce comparable improvements in posterior chain flexibility in physically active young adults. Therefore, the primary aim of the present study was to evaluate the effectiveness of a 120 s intervention consisting of static stretching and full range-of-motion resistance training in improving range of motion among physically active young adults.

## 2. Materials and Methods

### 2.1. Ethical Considerations

**Figure 1:**
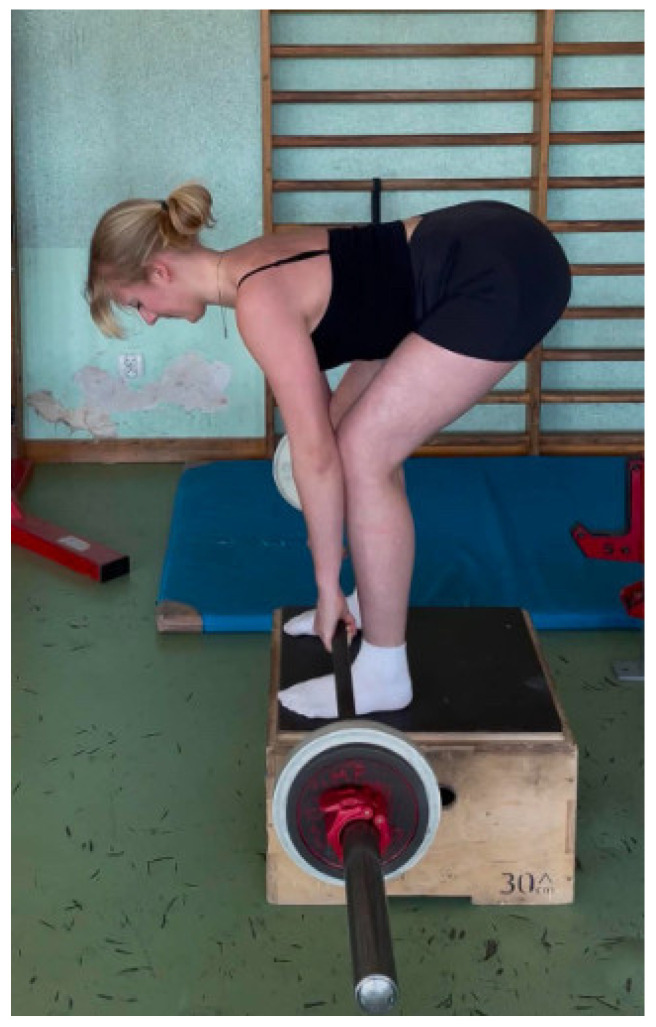
End position of the Romanian deadlift (RDL).

The research was approved by the Senate Research Ethics Committee of the Jozef Pilsudski University of Physical Education in Warsaw (SKE.0030.59.2025) and carried out in accordance with the principles outlined in the Declaration of Helsinki. The study was retrospectively registered at ClinicalTrials.gov (identification number: NCT07061041; registration date: 10 July 2025). Written informed consent for publication of the image was obtained from the participant shown in Figure 1.

### 2.2. Study Design

A three-arm randomized controlled trial with pre- and post-intervention assessments was conducted to evaluate the effects of static stretching and full range-of-motion resistance training on posterior chain flexibility following a six-week intervention. Participants were recruited and enrolled by the principal investigator. Following eligibility screening and baseline assessment, participants were randomly allocated in a 1:1:1 ratio to static stretching, resistance training or control. The randomization sequence was generated before participant enrollment by the principal investigator using a computer-generated randomization procedure. No stratification or blocking procedures were applied. The principal investigator recruited and enrolled participants and assigned them to the study groups according to the allocation sequence. The allocation sequence was accessible to the investigator responsible for enrollment before group assignment; therefore, allocation concealment was not implemented. This lack of allocation concealment may have increased the risk of selection bias and should be considered when interpreting the findings. Allocation concealment was not implemented because the study was conducted at a single academic center with limited personnel resources, making independent allocation concealment infeasible. Each group consisted of 16 participants. At the end of the intervention period, participants completed a post-test using the same flexibility assessment as in the pre-test to evaluate changes in range of motion. Participant blinding was not implemented. The absence of participant blinding was attributable to the nature of the intervention, as individuals performing stretching or resistance training exercises are inherently aware of both the type and purpose of the activity. Familiarization sessions were not conducted for the SS group because the applied flexibility tasks are simple, low-skill movements that are minimally influenced by motor learning or technical proficiency. Previous studies have demonstrated that familiarization sessions do not improve the reliability of posterior chain flexibility assessments when using commonly employed measures such as the stand-and-reach and sit-and-reach tests, supporting the decision to omit such sessions [24,25,26]. However, it should be emphasized that this evidence was derived from school-aged populations rather than adults, and therefore its applicability to the present sample should be interpreted with caution. Participants allocated to the RT group completed two familiarization sessions before the intervention to standardize exercise execution and to minimize potential learning effects during the study period. However, it should be noted that the absence of comparable familiarization sessions in the SS group resulted in unequal contact time with the researchers between the intervention groups, which may have introduced a potential risk of performance or attention bias.

### 2.3. Participants

The study applied the following eligibility criteria: participants were required to be ≥18 years of age and free from musculoskeletal injuries or surgical procedures involving the lower extremities or trunk within the previous six months. Individuals who did not meet these criteria were excluded from participation. A total of 54 individuals initially consented to participate in the study. During the intervention period, six participants were lost due to non-compliance with the intervention protocol, resulting in a dropout rate of 11.1%. Statistical analyses were performed according to the per-protocol principle. Only participants who completed the intervention and post-intervention assessment were included in the final analysis. Consequently, data from 48 participants were included in the final analysis and allocated to three groups. The static stretching group (SS) consisted of 16 participants, including an equal number of females and males, whereas the resistance training group (RT) included 16 participants (7 women and 9 men). The control group (CG) consisted of 16 participants, with an equal distribution of females and males. All participants were physically active, and their level of physical activity was classified according to the widely adopted framework proposed by McKay et al. [27]. Based on this classification, participants were categorized as Tier 1. Tier 1 includes individuals who meet the World Health Organization recommendations for physical activity and regularly engage in multiple forms of exercise. Participants were between 18 and 25 years of age. Prior to enrollment, all individuals completed a screening questionnaire designed to collect basic demographic information and self-reported physical activity data. The questionnaire included items regarding full name, optional e-mail address (for participants interested in receiving the study results), body height (cm), body mass (kg), and physical activity level.

### 2.4. Pre-Test and Post-Test Procedures

**Figure 2:**
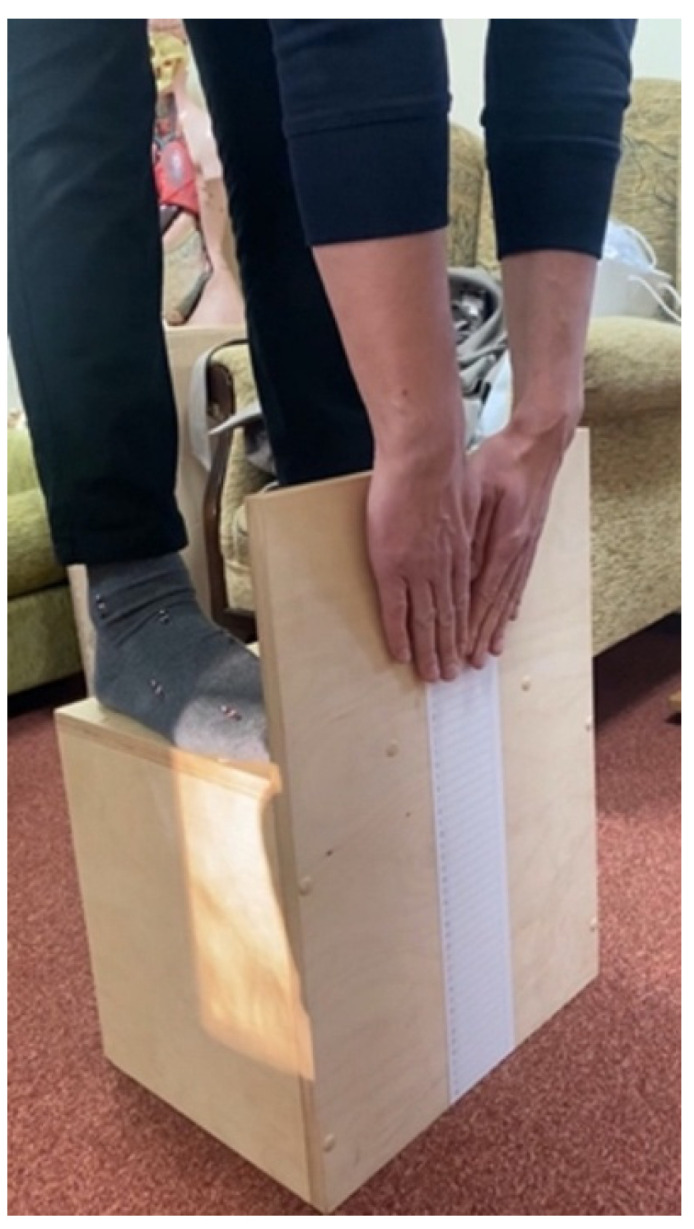
End position of the stand-and-reach test on the measuring platform.

Flexibility was assessed using a modified stand-and-reach test, a reliable and widely used method for evaluating posterior chain flexibility, with previous studies reporting high test–retest reliability [28]. Participants stood with their feet positioned hip-width apart and their knees fully extended before performing a forward bend, reaching as far as possible while maintaining knee extension (Figure 2). Flexibility was assessed using a custom measurement box that allowed participants to reach below foot level. A calibrated centimeter scale was attached to the top surface of the box, positioned perpendicular to the participant’s body axis and extending 25 cm beyond the front edge to facilitate accurate measurement of fingertip reach distance. Each participant performed two trials, and the mean value was used for statistical analysis.

The same assessment was repeated following the 6-week intervention period to evaluate changes in posterior chain flexibility. The post-intervention measurements were conducted by an assessor who was trained in the testing procedure and blinded to group allocation, with no knowledge of whether participants belonged to the intervention or control groups.

### 2.5. Intervention Protocol

The intervention lasted 6 weeks and consisted of one supervised training session per week for both the resistance training (RT) and static stretching (SS) groups. Before each intervention session, participants in both intervention groups performed the same standardized warm-up, consisting of 2 min of running followed by two sets of single-leg glute bridges. Each set consisted of 8–12 repetitions per limb and was performed at an RPE of 8. A 60 s rest interval was provided between sets. The load was adjusted individually; if a participant was able to perform more than 12 repetitions at the prescribed RPE, the load was increased during the subsequent session to maintain the target intensity. Importantly, the single-leg glute bridge was not performed through a full range of motion. Across the 6-week intervention period, each participant completed a total of 12 min of running and 12 sets of single-leg glute bridges per limb, corresponding to 96–144 repetitions per limb.

Following the warm-up, participants in the SS group performed two sets of static stretching using a forward-bending exercise while maintaining full knee extension throughout the movement. Each set lasted 60 s, with a 45 s interval between sets. Participants in the SS group were instructed to maintain a high stretching intensity, defined as the maximal tolerable level of discomfort, during every training session. Consequently, if flexibility improved over the course of the intervention, participants naturally increased their stretching range to maintain the same relative intensity (i.e., the maximal point of tolerable discomfort) at their newly achieved range of motion. The required intensity level was reinforced before each session. For the RT group, a rest interval of 120 s was used between sets. The initial training load was individually determined during the familiarization sessions to allow participants to complete 3 sets of 10 repetitions at an intensity corresponding to RPE 8, approximately 2 repetitions in reserve. Training load was progressed throughout the intervention whenever a participant was able to complete the prescribed number of repetitions while reporting an RPE lower than 8 or more than 2 repetitions in reserve. Initial and final external loads were recorded for each participant and reported descriptively as mean ± SD for the RT group. Following the warm-up, participants in the RT group performed Romanian deadlifts (RDLs) through a full range of motion for 3 sets of 10 repetitions (Figure 1). Each repetition consisted of a 3 s eccentric phase and a 1 s concentric phase. Romanian deadlifts were selected because they represent a fundamental multi-joint exercise targeting the posterior chain and are widely incorporated into strength and conditioning programs. Their widespread use makes them an appropriate model for investigating whether a commonly prescribed resistance exercise can also contribute to improvements in posterior chain flexibility, thereby providing an additional functional benefit without increasing overall training time. Participants were instructed to maintain their habitual training routines throughout the study period. They were specifically asked not to introduce any additional stretching exercises or full range-of-motion resistance training targeting the posterior chain muscles. In the event of any changes to their physical activity routines, participants were required to inform the research team. Individuals assigned to the control group continued their usual physical activity and habitual training routines, which did not include static stretching exercises or full range-of-motion posterior chain resistance training. Participants were instructed to report any symptoms or complaints that they believed might be related to the intervention. Adverse events were defined as any pain or discomfort affecting the posterior chain in the static stretching group and any pain or discomfort affecting any region of the body in the resistance training group.

### 2.6. Statistical Analysis

Data from both the pre-test and post-test assessments were analyzed. Statistical analyses were performed using Statistica software (version 14; TIBCO Software Inc., Palo Alto, CA, USA). The normality of the analyzed variables was assessed using the Shapiro–Wilk test. Descriptive statistics are presented as means and standard deviations, with 95% confidence intervals where appropriate. Baseline differences between the control group (CG), static stretching group (SS), and resistance training group (RT) were assessed using one-way analysis of variance (ANOVA). This analysis was used to compare baseline demographic and anthropometric characteristics, including age, body height, body mass, and BMI, as well as baseline flexibility.

The primary analysis was conducted using the per-protocol population, defined as participants who completed both baseline and post-intervention outcome assessments. The primary analysis was conducted using a complete-case approach and included participants with available baseline and post-intervention outcome data. Six randomized participants withdrew after baseline testing but before attending any intervention session and therefore had no post-intervention outcome data. A complete-case approach was considered appropriate because the proportion of missing outcome data was relatively small, all withdrawals occurred before exposure to the allocated interventions, and the withdrawals were not related to intervention effectiveness, tolerability, or adverse events. The primary analysis of the intervention effect was performed using a 3 × 2 mixed-model repeated-measures ANOVA, with group (CG, SS, RT) entered as the between-subject factor and time (pre-test, post-test) entered as the within-subject factor. This model was used to evaluate the main effect of group, the main effect of time, and, most importantly, the Group × Time interaction, which indicated whether changes in posterior chain flexibility differed between groups over time. Following a significant Group × Time interaction, pairwise comparisons of pre–post change scores were performed to identify between-group differences in the magnitude of change. The planned pairwise comparisons included CG vs. SS, CG vs. RT, and SS vs. RT. To account for multiple comparisons, the resulting *p*-values were adjusted using the Holm-Bonferroni procedure. All *p*-values reported for these pairwise comparisons are Holm-Bonferroni-adjusted values. Pre-post change scores were calculated as post-test minus pre-test and are presented as unadjusted descriptive follow-up information to aid interpretation of the interaction effect. Because a numerical difference in baseline flexibility was observed between groups and a significant between-group difference in age was present at baseline, an additional analysis of covariance (ANCOVA) was performed. In this model, post-intervention flexibility was entered as the dependent variable, group was entered as the fixed factor, and baseline flexibility and age were included as covariates. This analysis was used to determine whether the effect of group remained significant after adjustment for baseline flexibility and age. To examine whether sex modified the intervention effect, a 3 × 2 × 2 mixed-model repeated-measures ANOVA was additionally performed, with group (CG, SS, RT) and sex (female, male) entered as between-subject factors and time (pre-test, post-test) entered as the within-subject factor. The Group × Time × Sex interaction was used to determine whether changes in posterior chain flexibility differed between groups depending on sex. Because of the small subgroup sample sizes, sex-stratified follow-up comparisons were interpreted as exploratory. All statistical tests were performed at a significance level of α = 0.05. Effect sizes for ANOVA and ANCOVA models were reported as partial eta squared (partial η^2^). Cohen’s d was used to describe the magnitude of selected pre-post intervention effects and was interpreted according to Cohen’s recommendations, where d = 0.20, 0.50, and 0.80 indicate small, medium, and large effects, respectively [29]. Within-group effect sizes for pre–post changes were calculated as Cohen’s d by dividing the mean pre–post difference by the pooled standard deviation of the pre- and post-intervention values. The pooled standard deviation was calculated from the pre- and post-intervention standard deviations. Ninety-five percent confidence intervals for within-group Cohen’s d were calculated using the standard error of the standardized mean change, taking into account the Pearson correlation between pre- and post-intervention measurements within each group. Between-group effect sizes for differences in pre–post change were calculated as Cohen’s d by dividing the mean difference in change scores between groups by the pooled standard deviation of the change scores. Ninety-five percent confidence intervals for between-group Cohen’s d were calculated using the standard error of Cohen’s d for independent groups, based on the effect size value and the sample sizes of the compared groups. The sample size was determined by feasibility, based on the number of participants available during the recruitment period. Therefore, the present study should be considered exploratory. A sensitivity analysis was performed using G*Power (version 3.1.9.7) to determine the minimum detectable effect size for the available sample. The analysis was conducted for F tests using repeated-measures ANOVA, within–between interaction, with three groups, two measurements, α = 0.05, power = 0.80, a conservative assumed correlation among repeated measures of 0.50, and a nonsphericity correction of ε = 1. The analysis indicated that the study was powered to detect a minimum effect size of f = 0.23 for the Group × Time interaction. Therefore, smaller effects may not have been detected. The observed correlation between repeated measures in the final analyzed sample was r = 0.92.

## 3. Results

**Figure 3:**
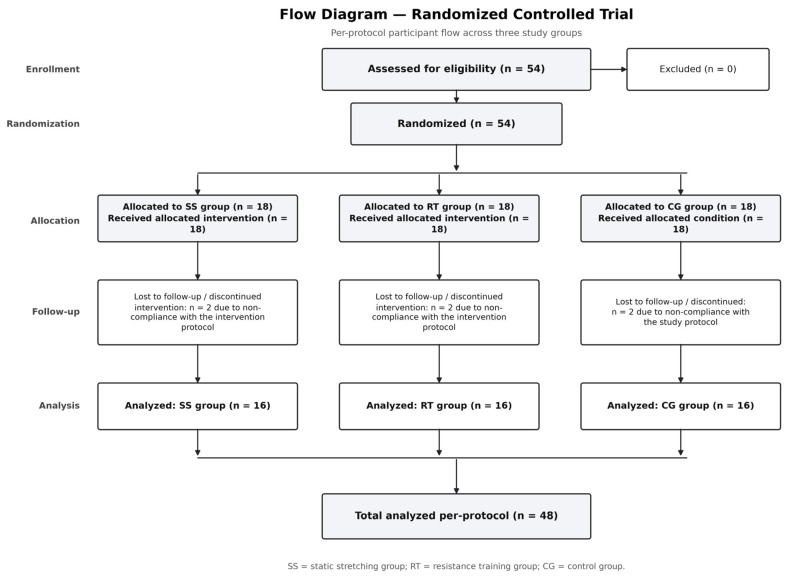
Participant flow diagram.

The participant flow throughout the study is presented in Figure 3. A total of 54 participants were assessed for eligibility and randomized into three groups: static stretching (SS), resistance training (RT), and control group (CG), with 18 participants allocated to each group. During the intervention period, two participants from the SS and two participants from the RT group discontinued participation due to non-compliance with the intervention protocol, whereas two participants from the CG were excluded due to non-compliance with the study protocol. Therefore, 48 participants completed the study and were included in the final per-protocol analysis. All participants included in the final analysis attended all scheduled intervention sessions. No adverse events or intervention-related complaints were reported during the study period. In the RT group, the initial external load was 42.6 ± 24.2 kg and increased to 58.9 ± 21.9 kg in the final week of the intervention.

Table 1 presents the descriptive characteristics of the control, static stretching, and resistance training groups. Neither intervention group differed significantly from the control group in terms of body height, body mass, or BMI. A significant between-group difference was observed for age (*p* < 0.05). The resistance training group differed significantly from the static stretching group (21.19 [±2.34] vs. 18.75 [±0.68]), and the control group (21.19 [±2.34] vs. 18.88 [±0.50]).

Table 2 presents the effects of the interventions on the selected outcome measures. The repeated-measures ANOVA revealed no significant main effect of group, F(2,45) = 1.51, *p* = 0.232, partial η^2^ = 0.063, but showed a significant main effect of time, F(1,45) = 30.46, *p* < 0.001, partial η^2^ = 0.404. Importantly, a significant Group × Time interaction was observed, F(2,45) = 13.24, *p* < 0.001, partial η^2^ = 0.370, indicating that changes in posterior chain flexibility differed between groups over time. Separate between-group comparisons showed no significant differences between groups in either the pre-test or post-test assessments. Baseline flexibility did not differ significantly between the control, static stretching, and resistance training groups, F(2,45) = 2.01, *p* = 0.146. However, given the numerical difference in pre-test flexibility between the SS and RT groups, an additional ANCOVA was performed to account for potential baseline imbalance. In this model, post-intervention flexibility was entered as the dependent variable, group was entered as the fixed factor, and baseline flexibility and age were included as covariates. The effect of group remained statistically significant after adjustment for baseline flexibility and age, F(2,43) = 11.98, *p* < 0.001, partial η^2^ = 0.358. Baseline flexibility was a significant covariate, F(1,43) = 385.73, *p* < 0.001, whereas age was not a significant covariate, F(1,43) = 0.095, *p* = 0.759. Follow-up comparisons of pre-post change scores showed a significantly greater improvement in the RT group compared with the CG (−0.25 [±0.93] vs. 1.81 [±1.75]; *p* = 0.039), as well as in the SS group compared with the CG (−0.25 [±0.93] vs. 3.93 [±3.45]; *p* < 0.001). No statistically significant difference in pre-post change was observed between the RT and SS groups (*p* > 0.05). Between-group effect sizes for differences in pre–post change were large for both intervention groups compared with the control group: RT vs. CG, Cohen’s d = 1.47, 95% CI: 0.65–2.29 and SS vs. CG, Cohen’s d = 1.65, 95% CI: 0.81–2.50. The between-group effect size for the difference in pre–post change between the SS and RT groups was moderate (Cohen’s d = 0.77, 95% CI: 0.02–1.52). Within-group effect sizes indicated a small improvement in the resistance training group (Cohen’s d = 0.30; 95% CI: 0.11–0.49) and a small, nearly moderate improvement in the static stretching group (Cohen’s d = 0.47; 95% CI: 0.19–0.75).

Table 3 presents the characteristics of the control, static stretching, and resistance training groups stratified by sex. No statistically significant differences were observed in age, body height, body mass, or BMI.

Table 4 presents the effects of the intervention on the analyzed outcome measures with respect to sex. A three-way mixed-model repeated-measures ANOVA was performed to examine whether sex modified the intervention effect. The analysis showed a significant Group × Time interaction, F(2,42) = 12.94, *p* < 0.001, partial η^2^ = 0.381, whereas the Group × Time × Sex interaction was not significant, F(2,42) = 0.72, *p* = 0.491, partial η^2^ = 0.033. This indicates that changes in posterior chain flexibility differed between groups over time, but these changes were not significantly moderated by sex. In exploratory sex-stratified follow-up comparisons, a statistically significant difference in pre–post change was observed between women in the control group and women in the static stretching group (−0.25 [±1.28] vs. 4.75 [±4.17]; *p* < 0.001). The within-group effect size for the pre–post change in women in the static stretching group was moderate (Cohen’s d = 0.53, 95% CI: 0.02–1.05). Given the non-significant Group × Time × Sex interaction, the exploratory sex-stratified follow-up comparisons should not be interpreted as evidence of a sex-specific intervention effect. No significant differences in pre-post change were found between women in the control and resistance training groups, nor between men in the control group and men in either intervention group. Likewise, no significant between-group differences were observed for either sex in the pre-test or post-test assessments.

## 4. Discussion

Scientific evidence indicates that resistance training can effectively improve range of motion [14,15,30]. However, relatively few studies have investigated the effectiveness of low-volume resistance training interventions aimed at increasing range of motion, particularly in comparison with static stretching. Therefore, the present study focused on evaluating the effectiveness of a low-volume resistance training intervention relative to a low-volume static stretching protocol. The intervention duration for both groups was 120 s once a week. Both experimental groups were supervised during the training sessions.

The present study demonstrated that a single weekly static stretching session consisting of two 60 s sets separated by a 45 s rest interval, performed over a six-week period, was effective in improving range of motion. The intervention resulted in a mean 15.2% improvement in range of motion in the static stretching group, corresponding to a statistically significant improvement between the pre- and post-intervention measurements (*p* < 0.001). The findings of the present study are consistent with those reported by Shadmehr et al., who observed a 12.6% increase in flexibility following a four-week intervention consisting of 10 training sessions performed every other day. Each session included three sets of 10 s of static stretching, resulting in a total weekly stretching volume of 60 to 90 s (two to three sessions per week) [31]. Similarly, Davis et al. reported a 38% improvement following a weekly static stretching volume of 90 s distributed across three weekly sessions, each consisting of 30 s of stretching. However, it should be emphasized that the intervention group in that study included only five participants [32]. To our knowledge, no further studies have examined the effectiveness of static stretching protocols with a total weekly duration below 120 s.

Scientific evidence suggests that full range-of-motion resistance training can effectively improve ROM. In a study by Potier et al. [30], the authors investigated the effects of progressive eccentric hamstring resistance training on passive knee extension range of motion. Participants performed training on a unilateral leg curl machine three times per week using a 1-repetition maximum (1RM) load, with each repetition consisting of a 5 s eccentric and a 5 s concentric phase. The intervention lasted eight weeks. Weekly training volume increased progressively from 90 s at the beginning of the intervention to 720 s during the final week. The resistance training group demonstrated a significant 5% increase in passive ROM as assessed using the passive knee extension (PKE) test. More recently, Rosenfeldt et al. [15] directly compared static stretching and full range-of-motion resistance training as methods for improving ROM. Both the resistance training and static stretching groups achieved comparable improvements in ROM. Importantly, the planned active exercise duration was matched between groups and increased progressively from 6 min and 24 s per week during the first week to 12 min and 48 s per week during the final week of the intervention. The findings of the aforementioned studies are consistent with the results obtained in the present investigation. However, it should be noted that the training volumes employed in those studies were substantially greater than that used in the current intervention. In the present study, the resistance training group demonstrated a mean improvement of 5.8% in posterior chain flexibility despite performing only 120 s of full range-of-motion resistance training once per week. At the same time, there is a lack of studies utilizing lower training volumes that could serve as a direct reference point for the findings reported here.

Despite growing evidence supporting the role of full range-of-motion resistance training in enhancing ROM, there is a lack of studies directly comparing low-volume resistance training with static stretching. Therefore, the present findings suggest that both static stretching performed for 120 s per week and full range-of-motion resistance training of the same duration are effective strategies for increasing ROM. These findings contribute to the growing body of evidence supporting the efficacy of low-volume interventions for flexibility development.

From a practical perspective, the observed improvements in reach distance should be interpreted in the context of the very low training volume used in the present study. Although the mean increase was numerically greater in the SS group than in the RT group (3.93 cm vs. 1.81 cm), both changes were achieved with only 120 s of supervised exercise per week. However, the practical importance of these changes remains uncertain, as the present study did not include a predefined criterion for determining whether the observed improvements were meaningful in functional or clinical terms. Therefore, the 1.81 cm improvement observed after full range-of-motion resistance training should be interpreted as a potential flexibility-related adaptation rather than definitive evidence of practical benefit. Similarly, the larger 3.93 cm improvement observed after static stretching suggests a greater numerical increase in posterior chain flexibility, but its functional relevance also requires further investigation. Future studies with larger sample sizes and predefined criteria for meaningful change are needed to better determine the practical significance of these findings.

A statistically significant difference in age was observed between groups, with the RT group being older than both the SS and CG groups. Although participants were randomly assigned to groups, such imbalance may occur in randomized trials with relatively small sample sizes, particularly when randomization is not stratified by age. This baseline imbalance should be acknowledged as a limitation of the present study. However, all participants were young adults and remained within a relatively narrow age range. To examine whether this imbalance influenced the study findings, an additional ANCOVA was performed with age included as a covariate. This analysis showed that age was not a significant covariate, F(1,43) = 0.095, *p* = 0.759, while the effect of group remained statistically significant after adjustment for age and baseline flexibility. Therefore, although the baseline age difference should be considered when interpreting the findings, the adjusted analysis did not indicate that age substantially influenced the observed changes in posterior chain flexibility.

Although the SS group demonstrated numerically greater improvements in posterior chain flexibility than the RT group (3.93 cm [±3.45 cm] vs. 1.81 cm [±1.75 cm]), no statistically significant between-group difference was observed (*p* > 0.05). This finding should be interpreted with caution, as the sensitivity analysis indicated that the present study was adequately powered to detect only large between-group effects. Consequently, the study may have lacked sufficient statistical power to detect small or moderate differences between the intervention groups. Future studies with larger sample sizes are warranted to determine whether a true difference exists between these interventions.

The small-to-moderate improvements observed in both the resistance training and static stretching groups (Cohen’s d = 0.30–0.53) may be explained by several factors. First, both intervention groups were small (*n* = 16), which limits the generalizability of the findings. Second, it should be acknowledged that a 120 s static stretching session is not directly equivalent to a 120 s full range-of-motion resistance training session. During resistance training, each repetition was performed with 3 s of eccentric and 1 s of concentric muscle action, whereas participants in the static stretching group maintained the stretched position continuously for 60 s during each set. Consequently, participants in the resistance training group accumulated less total time under stretch than those in the static stretching group. Given the relatively small sample size, the findings should therefore be interpreted with caution.

The unequal familiarization procedures between the intervention groups should be acknowledged. The RT group completed two familiarization sessions, whereas the SS group did not. However, these sessions were not intended to provide an additional training stimulus. Rather, they were devoted to standardizing Romanian deadlift technique, ensuring participant safety, and determining the appropriate training load. Although this procedure was necessary due to the technical demands of the Romanian deadlift, it resulted in greater contact time with the researchers in the RT group. Therefore, a potential performance or attention bias cannot be excluded and should be considered when interpreting the findings.

The lack of allocation concealment and participant blinding represents an important methodological limitation. These factors may have increased the risk of selection and performance bias and should therefore be considered when interpreting the findings. However, participant blinding was not feasible due to the nature of the interventions, as participants were aware of whether they were performing static stretching or resistance training. Future studies should use concealed allocation procedures and maintain blinded outcome assessment whenever possible to minimize bias.

## 5. Limitations

This study has both strengths and limitations. The major strengths include the randomized study design and the fact that every training session in both intervention groups was conducted under direct supervision, ensuring high protocol adherence and minimizing variability in intervention delivery. In addition, the planned active exercise duration was matched across the intervention groups, allowing for a more robust comparison of the effects of static stretching and full range-of-motion resistance training on posterior chain flexibility outcomes. The primary limitation of this study is the relatively small sample size, particularly for the sex-specific subgroup analyses. Therefore, these findings should be interpreted with caution and confirmed in studies with larger samples. An additional limitation of the study is the statistically significant difference in age between the RT group and both the SS and CG groups. Although participants were randomly allocated to the study groups, this baseline imbalance most likely resulted from the relatively small sample size within each group. However, given the small sample size, the results should be interpreted with caution and confirmed in studies involving larger cohorts. An additional limitation of the study is the lack of control over participants’ physical activity levels, relying instead on their self-reported declaration that static stretching and full range of motion resistance training were not included in their regular training routine. Another methodological limitation is that allocation concealment and participant blinding were not implemented, which may have increased the risk of selection and performance bias. Future studies should use concealed allocation procedures and, where feasible, blinded outcome assessment and other strategies to minimize bias. A further limitation is that the modified stand-and-reach test is a compound functional movement and does not isolate hamstring flexibility. The final reach distance may also be influenced by lumbopelvic and spinal mobility, scapular movement, and shoulder girdle positioning. Therefore, the results should be interpreted as changes in functional posterior chain flexibility rather than isolated changes in a single muscle group. More specific assessments, such as the passive straight leg raise test, would allow a more precise evaluation of hamstring flexibility. A further limitation is that both intervention groups, but not the control group, performed a standardized active warm-up before each session. Consequently, the improvements observed in the SS and RT groups relative to the control group cannot be attributed exclusively to static stretching or Romanian deadlifts, respectively, but should be interpreted as the effects of the complete intervention protocols. The use of a per-protocol analysis should be considered a limitation of the present study. A complete-case analysis was used because six participants withdrew after baseline testing but before commencing the allocated interventions and therefore did not provide post-intervention outcome data. Although these withdrawals occurred before intervention exposure and were not related to adverse events or intervention tolerability, excluding participants with missing outcome data may have introduced attrition bias and partially reduced the benefits of randomization. Another limitation is the unequal familiarization procedure between the intervention groups. The RT group completed two familiarization sessions to ensure proper exercise technique and load selection, whereas the SS group did not undergo familiarization because the stretching protocol was technically simple. However, this difference resulted in greater researcher contact time in the RT group and may have introduced attention bias.

## 6. Conclusions

In conclusion, the present study suggests that both static stretching and full range-of-motion resistance training, performed at a low weekly volume of 120 s under supervised conditions, may be effective strategies for improving posterior chain flexibility in physically active young adults. Furthermore, supervised time-efficient flexibility interventions may also be of considerable practical value for physically active individuals, who must integrate flexibility training with resistance training, sport-specific practice, conditioning, and recovery; however, this potential practical value should be confirmed in future studies.

## Figures and Tables

**Table 1:** Descriptive characteristics of the study groups.

	Control Group (*n* = 16)	Static Stretching Group (*n* = 16)	Resistance Training Group (*n* = 16)
Age (years ± SD)	18.88 [±0.50]	18.75 [±0.68]	21.19 [±2.34] *
Body height (cm ± SD)	173.00 [±6.15]	176.00 [±12.04]	175.20 [±8.53]
Body mass (kg ± SD)	66.25 [±10.61]	71.38 [±14.05]	72.19 [±10.98]
BMI (kg/m^2^ ± SD)	22.03 [±2.44]	22.81 [±2.35]	23.42 [±2.38]

cm—centimeters; SD—standard deviation; kg—kilograms; * *p* < 0.05.

**Table 2:** Effect of the intervention on the study groups. Values are presented as unadjusted descriptive statistics.

Group	Pre-Test ± SD (cm)	95% CI	Post-Test ± SD (cm)	95% CI	Change from Pre-Test to Post-Test ± SD (cm)	95% CI
Control group (*n* = 16)	28.88 ± 6.51	25.41–32.35	28.63 ± 6.60	25.11–32.15	−0.25 ± 0.93	−0.75–0.25
Static stretching group (*n* = 16)	25.94 ± 8.77	21.27–30.61	29.88 ± 7.97	25.63–34.13	3.93 ± 3.45 **^,†^	2.09–5.77
Resistance training group (*n* = 16)	31.06 ± 6.23	27.74–34.38	32.88 ± 5.93	29.72–36.04	1.81 ± 1.75 *^,†^	0.88–2.74

cm—centimeters, SD—standard deviation, pre-test—measurement before the intervention, post-test—measurement after the intervention, change from pre-test to post-test—difference between the pre- and post-intervention measurements, 95% CI—Confidence Interval 95%, * *p* < 0.05, ** *p* < 0.001, Cohen’s d: ^†^ −0.2–0.5.

**Table 3:** Descriptive characteristics of the study groups with respect to sex. Values are presented as unadjusted descriptive statistics.

	Control Group (*n* = 16)		Static Stretching Group (*n* = 16)		Resistance Training Group (*n* = 16)	
	Female (*n* = 8)	Male (*n* = 8)	Female (*n* = 8)	Male (*n* = 8)	Female (*n* = 7)	Male (*n* = 9)
Age (years ± SD)	18.88 (±0.35)	18.88 (±0.64)	18.63 (±0.74)	18.88 (±0.64)	20.43 (±1.99)	21.78 (±2.54)
Body height (cm ± SD)	168.25 (±4.30)	177.00 (±4.04)	168.29 (±8.18)	185.00 (±5.66)	167.14 (±2.67)	181.44 (±5.52)
Body mass (kg ± SD)	57.75 (±4.74)	74.75 (±7.32)	60.88 (±10.13)	81.88 (±8.25)	61.86 (±3.86)	80.22 (±7.00)
BMI (kg/m^2^ ± SD)	20.35 (±2.06)	23.86 (±1.51)	21.85 (±1.96)	23.71 (±2.41)	22.14 (±1.24)	24.41 (±2.63)

cm—centimeters, SD—standard deviation, kg—kilograms.

**Table 4:** Effect of the intervention on the study groups with respect to sex.

Group	Pre-Test ± SD (cm)	95% CI	Post-Test ± SD (cm)	95% CI	Change from Pre-Test to Post-Test ± SD (cm)	95% CI
Control group, female (*n* = 8)	30.00 ± 6.30	24.82–35.18	29.75 ± 6.58	24.42–35.08	−0.25 ± 1.28	−1.32–0.82
Control group, male (*n* = 8)	27.75 ± 6.94	21.87–33.63	27.50 ± 6.87	21.65–33.35	−0.25 ± 0.46	−0.64–0.14
Static stretching group, female (*n* = 8)	25.88 ± 9.14	18.03–33.72	30.63 ± 8.70	23.04–38.21	4.75 ± 4.17 *^,‡^	1.18–8.32
Static stretching group, male (*n* = 8)	26.00 ± 9.01	18.11–33.89	29.13 ± 7.68	22.70–35.55	3.13 ± 2.59	0.74–5.51
Resistance training group, female (*n* = 7)	31.29 ± 5.74	25.77–36.80	33.00 ± 5.35	27.79–38.21	1.71 ± 1.25	0.56–2.87
Resistance training group, male (*n* = 9)	30.89 ± 6.94	25.55–36.23	32.78 ± 6.67	27.83–37.72	1.89 ± 2.15	0.28–3.50

cm—centimeters, SD—standard deviation, pre-test—measurement before the intervention, post-test—measurement after the intervention, change from pre-test to post-test—difference between the pre- and post-intervention measurements, 95% CI—Confidence Interval 95%, * *p* < 0.05, Cohen’s d: ^‡^ −0.5–0.8.

## Data Availability

The data are available from the corresponding author upon reasonable request. The data are not publicly available due to privacy restrictions.

## References

[1] Konrad A., Nakamura M., Paternoster F.K., Tilp M., Behm D.G. (2022). A comparison of a single bout of stretching or foam rolling on range of motion in healthy adults. Eur. J. Appl. Physiol..

[2] Ingram L.A., Tomkinson G.R., d’Unienville N.M.A., Gower B., Gleadhill S., Boyle T., Bennett H. (2025). Mechanisms underlying range of motion improvements following acute and chronic static stretching: A systematic review, meta-analysis and multivariate meta-regression. Sports Med..

[3] Shah R., Samuel M.W., Son J. (2023). Acute and chronic effects of static stretching on neuromuscular properties: A meta-analytical review. Appl. Sci..

[4] Takeuchi K., Nakamura M., Fukaya T., Konrad A., Mizuno T. (2023). Acute and long-term effects of static stretching on muscle-tendon unit stiffness: A systematic review and meta-analysis. J. Sports Sci. Med..

[5] Panidi I., Donti O., Konrad A., Dinas P.C., Terzis G., Mouratidis A., Gaspari V., Donti A., Bogdanis G. (2023). Muscle architecture adaptations to static stretching training: A systematic review with meta-analysis. Sports Med.-Open.

[6] Freitas S.R., Mendes B., Le Sant G., Andrade R.J., Nordez A., Milanovic Z. (2018). Can chronic stretching change the muscle-tendon mechanical properties? A review. Scand. J. Med. Sci. Sports.

[7] Blazevich A.J., Cannavan D., Waugh C.M., Fath F., Miller S.C., Kay A.D. (2012). Neuromuscular factors influencing the maximum stretch limit of the human plantar flexors. J. Appl. Physiol..

[8] Thomas E., Bianco A., Paoli A., Palma A. (2018). The relation between stretching typology and stretching duration: The effects on range of motion. Int. J. Sports Med..

[9] Jafari B., Minoojejad H., Sheikhhoseini R., Rajabi R. (2025). Effect of remote myofascial intervention on musculoskeletal health and functional performance: A systematic review. Adv. Rehabil..

[10] Rajabi R., Khorramroo F., Mousavi S.H., Hernandez M.E. (2025). Virtual reality exercises targeting spinal alignment: A systematic review and meta-analysis. Biomed. Hum. Kinet..

[11] Medeiros D.M., Martini T.F. (2018). Chronic effect of different types of stretching on ankle dorsiflexion range of motion: Systematic review and meta-analysis. Foot.

[12] Konrad A., Alizadeh S., Daneshjoo A., Anvar S.H., Graham A., Zahiri A., Goudini R., Edwards C., Scharf C., Behm D.G. (2024). Chronic effects of stretching on range of motion with consideration of potential moderating variables: A systematic review with meta-analysis. J. Sport Health Sci..

[13] Ingram L.A., Tomkinson G.R., d’Unienville N.M.A., Gower B., Gleadhill S., Boyle T., Bennett H. (2025). Optimising the dose of static stretching to improve flexibility: A systematic review, meta-analysis and multivariate meta-regression. Sports Med..

[14] Afonso J., Ramirez-Campillo R., Moscão J., Rocha T., Zacca R., Martins A., Milheiro A.A., Ferreire J., Sarmento H., Clemente F.M. (2021). Strength training versus stretching for improving range of motion: A systematic review and meta-analysis. Healthcare.

[15] Rosenfeldt M., Stien N., Behm D.G., Saeterbakken A.H., Andersen V. (2024). Comparison of resistance training vs static stretching on flexibility and maximal strength in healthy physically active adults: A randomized controlled trial. BMC Sports Sci. Med. Rehabil..

[16] Elgaddal N., Kramarow E.A., Reuben C. (2022). Physical activity among adults aged 18 and over: United States, 2020. NCHS Data Briefs.

[17] Ministry of Sport and Tourism (2024). Physical Activity Level of Poles 2024: Quantitative Research Report.

[18] Schutzer K.A., Graves B.S. (2004). Barriers and motivations to exercise in older adults. Prev. Med..

[19] Burton E., Farrier K., Lewin G., Pettigrew S., Hill A.M., Airey P., Bainbridge L., Hill K.D. (2017). Motivators and barriers for older people participating in resistance training: A systematic review. J. Aging Phys. Act..

[20] Fyfe J.J., Hamilton D.L., Daly R.M. (2022). Minimal-dose resistance training for improving muscle mass, strength, and function: A narrative review of current evidence and practical considerations. Sports Med..

[21] Little J.P., Langley J., Lee M., Myette-Côté E., Jackson G., Durrer C., Gibala M.J., Jung M.E. (2019). Sprint exercise snacks: A novel approach to increase aerobic fitness. Eur. J. Appl. Physiol..

[22] Jenkins E.M., Nairn L.N., Skelly L.E., Little J.P., Gibala M.J. (2019). Do stair climbing exercise “snacks” improve cardiorespiratory fitness?. Appl. Physiol. Nutr. Metab..

[23] Yin M., Deng S., Chen Z., Zhang B., Zheng H., Bai M., Li H., Zhang X., Deng J., Liu Q. (2024). Exercise snacks are a time-efficient alternative to moderate-intensity continuous training for improving cardiorespiratory fitness but not maximal fat oxidation in inactive adults: A randomized controlled trial. Appl. Physiol. Nutr. Metab..

[24] Vrbik I., Sporiš G., Štefan L., Madić D., Trajković N., Valantine I., Milanović Z. (2017). The influence of familiarization on physical fitness test results in primary school-aged children. Pediatr. Exerc. Sci..

[25] Coledam D.H.C., de Oliveira R.D.C. (2020). Assessment of physical fitness among non-athlete adolescents: Effect of familiarization sessions. Balt. J. Health Phys. Act..

[26] Međedović B., Romanov R., Zubanov V., Perić D., Stupar D., Ahmetović Z. (2018). Influence of familiarization on preschool children’s motor tests results. Acta Gymnica.

[27] McKay A.K.A., Stellingwerff T., Smith E.S., Martin D.T., Mujika I., Goosey-Tolfrey V.L., Sheppard J., Burke L.M. (2022). Defining training and performance caliber: A participant classification framework. Int. J. Sports Physiol. Perform..

[28] Perret C., Poiraudeau S., Fermanian J., Lefèvre Colau M.M., Mayoux Benhamou M.A., Revel M. (2001). Validity, reliability, and responsiveness of the fingertip-to-floor test. Arch. Phys. Med. Rehabil..

[29] Brydges C.R. (2019). Effect size guidelines, sample size calculations, and statistical power in gerontology. Innov. Aging.

[30] Potier T.G., Alexander C.M., Seynnes O.R. (2009). Effects of eccentric strength training on biceps femoris muscle architecture and knee joint range of movement. Eur. J. Appl. Physiol..

[31] Shadmehr A., Hadian M.R., Naiemi S.S., Jalaie S. (2009). Hamstring flexibility in young women following passive stretch and muscle energy technique. J. Back Musculoskelet. Rehabil..

[32] Davis D.S., Ashby P.E., McCale K.L., McQuain J.A., Wine J.M. (2005). The effectiveness of 3 stretching techniques on hamstring flexibility using consistent stretching parameters. J. Strength Cond. Res..

